# Pyuria as a Predictive Marker of Bacillus Calmette–Guérin Unresponsiveness in Non-Muscle Invasive Bladder Cancer

**DOI:** 10.3390/jcm10173764

**Published:** 2021-08-24

**Authors:** Jungyo Suh, Hyeong Dong Yuk, Chang Wook Jeong, Cheol Kwak, Hyeon Hoe Kim, Ja Hyeon Ku

**Affiliations:** 1 Department of Urology, Asan Medical Centre, Seoul 05505, Korea; uro_jun@amc.seoul.kr; 2 Department of Urology, Seoul National University Hospital, Seoul 03080, Korea; armenia8@snu.ac.kr (H.D.Y.); drboss@snu.ac.kr (C.W.J.); mdrafael@snu.ac.kr (C.K.); hhkim@snu.ac.kr (H.H.K.); 3 Department of Urology, Seoul National University College of Medicine, Seoul 03080, Korea

**Keywords:** pyuria, non-muscle invasive bladder cancer, BCG immunotherapy

## Abstract

This study aims to investigate the clinical role of preoperative pyuria for predicting bacillus Calmette–Guérin (BCG) unresponsiveness in non-muscle invasive bladder cancer (NMIBC). We performed a logistic regression analysis on 453 patients with NMIBC who were treated with BCG immunotherapy after a transurethral resection of bladder tumours, to evaluate predictive factors of BCG unresponsiveness. We also analysed univariate and multivariable survival data to estimate the prognostic impact of pyuria. Of the total study population, 37.6% (170/453) of patients had BCG unresponsiveness. A multivariable logistic regression analysis revealed that a history of upper urinary tract cancer (odds ratio (OR): 1.86, 95% confidence interval (CI): 1.04–3.32, *p*-value = 0.035) and the presence of pyuria (OR: 1.51, 95% CI: 1.01–2.27, *p* = 0.047) and tumour multiplicity (OR: 1.80, 95% CI: 1.18–2.75, *p*-value < 0.001) were significant predictors of BCG unresponsiveness. A Cox proportional hazards analysis model showed that pyuria was a significant prognostic factor for progression-free survival (hazard ratio: 4.51, 95% CI: 1.22–16.66, *p* = 0.024). A history of upper urinary tract cancer and the presence of pyuria and tumour multiplicity are predictive markers of BCG unresponsiveness. For patients with NMIBC who have preoperative pyuria, treatment using BCG should be considered cautiously.

## 1. Introduction

Bladder cancer is the 10th most common cancer worldwide and is highly prevalent in developed countries [1]. Up to 75% of patients with bladder cancer initially present with non-muscle invasive bladder cancer (NMIBC); however, 70% and 30% of patients with NMIBC experience recurrence and progression, respectively, within 5 years. Treatment with bacillus Calmette–Guérin (BCG) immunotherapy is recommended for patients with NMIBC, for whom there is an intermediate-to-high risk of recurrence or progression [2,3].

Although most patients with NMIBC initially respond to BCG immunotherapy, some experience recurrence or progression during or after treatment. ‘BCG unresponsive’ is a complex term that refers to a heterogeneous groups of patients for whom treatment with BCG immunotherapy is not recommended [4]. This term also describes patients in whom high-grade cancer persists even after they receive BCG treatment (‘BCG refractory’) and patients who experience recurrence after reaching a disease-free state (‘BCG relapsing’) within 6 months of their last BCG exposure [4]. The only recommended treatment for BCG-unresponsive patients with NMIBC is radical cystectomy [2,3]. Despite their importance, predictors of BCG unresponsiveness have not been clearly investigated [5].

More than 40 years after its initial clinical application, the mechanism of BCG immunotherapy remains unclear. Current evidence suggests that a BCG-mediated local inflammatory response leads to a therapeutic effect [6]. Paradoxically, a tumour inflammatory response is itself known to be a poor prognostic marker for bladder cancer [7,8]. Recently, the clinical role of pyuria as a non-invasive biomarker for bladder cancer recurrence and progression was highlighted by a Japanese group [9]. In this study, we evaluated the predictive impact of preoperative pyuria on BCG unresponsiveness in intermediate-to-high-risk NMIBC, using a prospectively collected dataset of the trans-urethral resection of bladder tumours (TURBT) cohort.

## 2. Materials and Methods

### 2.1. Ethics Approval and Informed Consent

This study was approved by the SNUH Institutional Review Board (SNUH IRB). Informed consent for the academic use of their clinical data was obtained from each patient at the time of their enrolment in the prospective registry (IRB No.: H-1506-122-682). For this ad hoc study, the SNUH IRB approved the academic use of registry data that focused on pyuria and BCG unresponsiveness, with respect to NMIBC that was treated with BCG (IRB No.: 2013-018-1202). All study processes were performed in accordance with relevant guidelines and regulations.

### 2.2. Patient Selection and Cohort Follow-Up Protocols

The Seoul National University Hospital Prospectively Enrolled Registry for Urothelial Carcinoma with Transurethral Resection of Bladder Tumor is a sub-cohort of a multidisciplinary, biobank-linked cohort of patients with genitourinary cancer in high-volume tertiary institutions [10]. Of 1557 patients with NMIBC who underwent TURBT between March 2016 and October 2020, the data from 453 patients treated with BCG-instillation therapy were collected for this analysis. To clarify the definition of ‘BCG unresponsiveness’, patients who received less than 6 months follow-up were excluded. Blood and urine laboratory tests were performed within 40 days of performing TURBT. All patients submitted a urine culture before TURBT. A patient displaying pyuria and febrile symptoms before TURBT was treated with antibiotics. All patients who had high-grade cancer during the initial TURBT underwent repeated TURBT within 1 month of the initial operation. NMIBC was confirmed after a review of the pathological specimens obtained from the initial or repeated TURBT, according to current guidelines [2,3].

BCG instillation and maintenance therapy were administered in accordance with the relevant guidelines. The BCG instillation protocol involved six cycles of weekly or biweekly induction and maintenance of BCG in tolerant patients. The maintenance schedule included intensive instillation every 3, 6 and 12 months in the first year and every 6 months for 2 additional years thereafter [11]. Cystoscopy and urine cytology were performed every 3 months to assess the recurrence of bladder cancer after any treatment cycle, including TURBT, and after induction and maintenance BCG. BCG unresponsiveness was defined as BCG refractory or BCG relapse with adequate BCG treatment [4]. Pyuria was defined as the presence of five or more white blood cells in a urine sample, as observed with a high-power field [12]. A tumour size of >3 cm was considered a large tumour.

### 2.3. Statistical Analyses

All continuous variables are described as mean ± standard deviation (interquartile range), whereas categorical variables are described as frequencies (percentages). Continuous variables were compared using Student’s *t*-test, and categorical variables were compared using a chi-squared test or Fisher’s exact test. Using univariate and multivariate logistic regression, we analysed the features associated with BCG unresponsiveness. The Kaplan–Meier curve and the log-rank test were used for a non-parametric comparison of RFS and PFS, relative to the presence of preoperative pyuria. For the multivariate analysis, the variables that showed a *p*-value of < 0.1 in the univariate analysis were selected. Statistical analyses were performed using Python version 3.9.0, based on packages dependent on SciPy [13]. Statistical significance was set at *p* < 0.05, and all reported *p*-values were two-sided.

## 3. Results

### 3.1. Patient Characteristics

**Figure 1:**
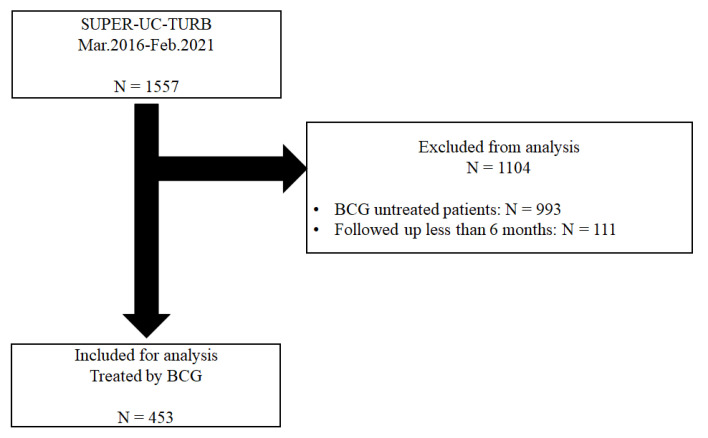
The flow chart of the inclusion and exclusion criteria and patient grouping based on the presence of pyuria before transurethral resection of the bladder.

Of the 1557 patients who underwent TURBT, 564 were treated with BCG immunotherapy. Finally, 453 patients who were treated with BCG immunotherapy after they had undergone TURBT at Seoul National University Hospital (SNUH) were analysed. A total of 174 patients had pyuria, and 262 patients had sterile urine before they underwent TURBT (Figure 1).

Of the 453 patients, 37.6% (170/453) were BCG unresponsive during the 25.0 ± 13.9 months of the follow-up period. A history of previous upper urinary tract cancer (UTUC) (16.5% vs. 9.9%, *p* = 0.056), pyuria (46.4% vs. 35.9%, *p* = 0.039) and tumour multiplicity (71.7% vs. 58.2%, *p* < 0.001) was significantly more prevalent in the BCG-unresponsive group than in the BCG-effective group (Table 1).

### 3.2. Univariate and Multivariate Logistic Regression for BCG Unresponsiveness

Through univariate analysis, a history of UTUC (odds ratio (OR): 1.80, 95% confidence interval (CI): 1.02–3.15, *p* = 0.041), the presence of pyuria (OR: 1.54, 95% CI: 1.04–2.29, *p* = 0.031), large tumour size (OR: 1.49, 95% CI: 1.01–2.21, *p* = 0.046) and the presence of multiple tumours (OR: 1.82, 95% CI: 1.20–2.75, *p* = 0.005) were associated with BCG unresponsiveness. The multivariate analysis revealed that a history of UTUC (OR: 1.86, 95% CI: 1.04–3.32, *p* = 0.035) and the presence of pyuria (OR: 1.51, 95% CI: 1.01–2.27, *p* = 0.047) and multiple tumours (OR: 1.80, 95% CI: 1.18–2.75, *p* < 0.001) were significant predictors of BCG unresponsiveness (Table 2).

### 3.3. Kaplan–Meier Curve with the Log-Rank Test for Recurrence- and Progression-Free Survival with or without Preoperative Pyuria in BCG-Treated Patients with Non-Muscle Invasive Bladder Cancer

**Figure 2:**
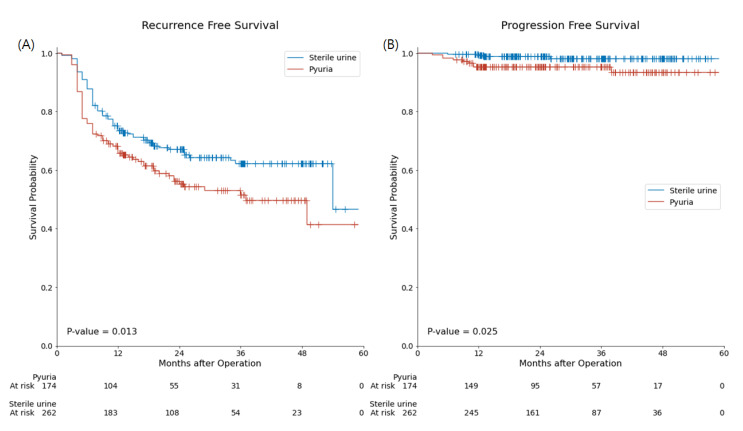
Kaplan–Meier curve and log-rank test for recurrence-free survival (**A**), and progression-free- survival (**B**) with or without preoperative pyuria in BCG-treated patients with non-muscle invasive bladder cancer. Redline and Blueline represent the pyuria group and sterile urine group, respectively.

In the sterile urine group, recurrence and progression occurred in 34.0% (89/262) and 1.5% (4/262) of patients, respectively. In the pyuria group, recurrence and progression were present in 44.3% (77/174) and 5.2% (9/174) of patients, respectively. Estimated recurrence-free survival (RFS) in the pyuria group was significantly shorter than in the sterile urine group (33.74 ± 2.07 months vs. 38.79 ± 1.51 months, respectively, *p* = 0.013). The calculated progression-free survival (PFS) in the pyuria group was also significantly shorter than that in the sterile urine group (55.40 ± 0.92 months vs. 56.70 ± 0.40 months, respectively, *p* = 0.025) (Figure 2).

### 3.4. Univariate and Multivariate Analysis Using the Cox Proportional Hazards Regression Model

Through the univariate analysis performed using the Cox proportional hazards regression model, with respect to RFS, the presence of pyuria (hazard ratio (HR): 1.47, 95% (CI): 1.08–2.00, *p* = 0.013), large tumour size (HR: 1.51, 95% CI: 1.11–2.06, *p* = 0.009) and the presence of multiple tumours (HR: 1.59, 95% CI: 1.13–2.24, *p* = 0.008) were found to be significant prognostic factors. Through the multivariate analysis performed using the Cox proportional hazards regression model, only the presence of multiple tumours was found to be a significant prognostic marker for RFS (HR: 1.51, 95% CI: 1.07–2.15, *p* = 0.019) (Table 3).

Through the univariate analysis performed using the Cox proportional hazards regression model, with respect to PFS, pathological T1 (HR: 3.33, 95% CI: 1.02–10.89, *p* = 0.046) and the presence of pyuria (HR: 3.52, 95% CI: 1.08–11.44, *p* = 0.036) were found to be statistically significant. The multivariate analysis using the Cox proportional hazards regression model showed that the presence of pyuria was the only significant prognostic marker for PFS (HR: 4.51, 95% CI: 1.22–16.66, *p* = 0.024) (Table 4).

## 4. Discussion

In this study, we evaluated the predictive markers of BCG unresponsiveness in NMIBC. In addition to other well-known predictive factors, such as a history of UTUC and the presence of multiple bladder tumours, pyuria is a predictive marker of BCG unresponsiveness. The Kaplan–Meier survival analysis showed a significantly worse RFS (33.74 ± 2.07 months vs. 38.79 ± 1.51 months, *p* = 0.013) and PFS (55.40 ± 0.92 months vs. 56.70 ± 0.40 months, *p* = 0.025) in the pyuria group than in the sterile urine group. The analysis using the Cox proportional hazards model showed that pyuria was a significant prognostic factor for PFS (HR: 4.51, 95% CI: 1.22–16.66, *p* = 0.024).

BCG immunotherapy was first used in 1979 for human bladder cancer and is still the most effective adjuvant treatment for managing NMIBC. However, up to 45% of patients do not respond to BCG immunotherapy within 2 years of initiating treatment [14]. The BCG-unresponsive patients show poorer oncological outcomes than the BCG-effective patients [15]. For this reason, early cystectomy is recommended for BCG-unresponsive patients over repeated BCG treatment [16]. In this study population, the rate of BCG unresponsiveness was 37.6% (170/453), which is similar to the results of previous studies [14].

Despite their clinical importance, the predictive markers of BCG unresponsiveness have not been adequately studied. Several clinicopathological parameters, including age, sex, tumour recurrence, tumour stage, tumour grade, the presence of carcinoma in situ and tumour multiplicities, are useful for predicting a patient’s BCG response [17]. However, BCG immunotherapy is recommended for high-grade, recurrent and multiple NMIBC tumours [2,3]; thus, in most cases, these clinical predictive factors cannot be used in real clinical practice. Recent studies found that urinary cytokine, fluorescent in situ hybridization (FISH) patterns of cytology [18], intra-cellular proteins such as nucleotide-binding oligomerization domain-like receptors (NLRs) [19] and tumour-infiltrating immune cells [20] are important biomarkers of BCG unresponsiveness. However, most of these biomarkers need additional procedures, like PCR, FISH Immunohistochemistry staining or Enzyme-Linked Immunosorbent Assay of a pathologic specimen or urine. In this study, we found that preoperative pyuria is an independent predictive marker of BCG unresponsiveness, which makes it an easy-to-use, cost-effective clinical parameter.

Miyake et al. compared an NMIBC with a history of UTUC cohort with a primary NMIBC cohort for BCG response in a propensity-matched population [21]. They reported that patients with NMIBC with a history of UTUC had a higher risk of bladder cancer recurrence than patients in the control group. A history of UTUC was identified as an independent prognostic factor of intravesical recurrence of urothelial cell carcinoma in a previous study [22,23]. These findings suggest that a history of UTUC increases the risk of bladder cancer recurrence or development, which supports this study’s finding that a history of UTUC is a predictive marker of BCG response.

In this study, preoperative pyuria was considered as a predictive marker of BCG unresponsiveness and a prognostic marker of PFS. Although the multivariate Cox analysis did not show pyuria to be a significant prognostic factor for RFS, the Kaplan–Meier analysis showed that the pyuria group had a significantly worse RFS compared to the sterile urine group. Several studies have reported preoperative pyuria as a prognostic marker of NMIBC recurrence [9,24]. The presence of pyuria was significantly higher in the BCG-unresponsive group than in the BCG-effective group (46.4% vs. 35.9%, *p* = 0.039) in this study, similar to previous reports [9,24]. However, these findings should be interpreted very carefully. The difference in the presence of preoperative pyuria was just 10% between the two groups (BCG unresponsive and BCG effective), suggesting that this finding is not clinically meaningful [25]. Similarly, although we found a statistically significant association between pyuria and PFS in this study, there was only a 1 month difference in the mean PFS between the two groups. Due to the long interval between the preoperative urine laboratory tests and recurrence or progression, preoperative pyuria itself may not have directly affected the oncological outcome of BCG-treated NMIBC. For this reason, we hypothesised that preoperative pyuria reflects disease aggressiveness [26] or a peritumoural inflammatory response [27], which can directly affect a BCG immunotherapy response. Therefore, we carefully considered preoperative pyuria as a predictive marker of BCG unresponsiveness that may indirectly affect RFS or PFS.

This study has several limitations. The definition of pyuria is not objective and varies in the literature [12,28]. Moreover, pyuria is associated with numerous conditions such as infection, autoimmune disease and TURBT itself [29]; thus, we need to interpret the results of this study cautiously. Despite these limitations, we successfully analysed the predictive markers of BCG unresponsiveness. To the best of our knowledge, this study investigated BCG unresponsiveness using the largest dataset to date, which was taken from a prospective cohort of patients with NMIBC. We identified a novel predictive marker of BCG unresponsiveness, namely, the presence of preoperative pyuria, and this finding was confirmed by survival analysis. A well-designed study with a more clearly selected cohort is needed to evaluate the prognostic impact of preoperative pyuria on RFS and PFS in patients with NMIBC.

In conclusion, the presence of pyuria, a history of UTUC and multiple tumours are predictive markers of BCG unresponsiveness. The preoperative pyuria group showed shorter RFS and PFS than the control group. The multivariate analysis performed using a Cox regression model showed that pyuria was a prognostic factor for PFS. For patients with NMIBC who have preoperative pyuria, treatment using BCG should be considered cautiously.

## Figures and Tables

**Table 1:** Patient demographics of renal insufficiency and control groups.

	BCG-Effective	BCG-Unresponsive	*p*-Value
Number of Patients	*N* = 283	*N* = 170	
Age, mean ± SD (IQR)	68.2 ± 9.8 (62.0–77.0)	67.7 ± 12.3 (62.0–76.0)	0.629
Previous TUR history, *n* (%)	68 (24.0)	52 (30.6)	0.155
Female, *n* (%)	41 (14.5)	23 (13.5)	0.885
Previous BCG treatment history, *n* (%)	23 (8.1)	20 (11.8)	0.266
Upper Tract Urothelial Carcinoma history, *n* (%)	28 (9.9)	28 (16.5)	0.056
Microscopic haematuria, *n* (%)	143 (52.8)	95 (57.2)	0.418
Pyuria, *n* (%)	97 (35.9)	77 (46.4)	0.039
BCG dose reduction, *n* (%)	11 (3.9)	7 (4.1)	0.882
Pathologic T stage, *n* (%)			0.950
Ta	162 (57.2)	96 (56.5)	
T1	121 (42.8)	74 (43.5)	
Tumour size (>3 cm), *n* (%)	97 (35.0)	74 (44.6)	0.057
Multiple tumours, *n* (%)	160 (58.2)	119 (71.7)	<0.001
Concomitant CIS, *n* (%)	63 (22.3)	37 (21.8)	0.995
Histologic variants, *n* (%)	13 (4.6)	7 (4.1)	0.998

**Table 2:** Univariate and multivariable logistic regression for BCG unresponsiveness.

	Univariate			Multivariable		
Variables	Odds Ratio	Confidence Interval	*p*-Value	Odds Ratio	Confidence Interval	*p*-Value
Age			0.799			
Age < 65	Ref.					
65 ≤ Age ≤ 75	0.89	0.51–1.54				
Age > 75	1.02	0.60–1.74				
Previous TUR history	1.39	0.91–2.13	0.126			
Sex (Female)	0.92	0.53–1.60	0.777			
Previous BCG treatment history	1.51	0.80–2.84	0.203			
Upper Tract Urothelial Carcinoma history	1.80	1.02–3.15	0.041	1.86	1.04–3.32	0.035
BCG dose reduction (half)	1.05	0.40–2.76	0.921			
Pathologic T stage (T1)	1.03	0.70–1.51	0.872			
Pyuria (more than W5)	1.54	1.04–2.29	0.031	1.51	1.01–2.27	0.047
Large tumour (3 cm)	1.49	1.01–2.21	0.046	1.39		0.136
Multiple tumour	1.82	1.20–2.75	0.005	1.80	1.18–2.75	<0.001
Concomitant CIS	0.97	0.61–1.54	0.902			

**Table 3:** Univariate and multivariable cox proportional hazards regression for recurrence-free survival.

	Univariate			Multivariable		
Variables	Hazard Ratio	Confidence Interval	*p*-Value	Hazard Ratio	Confidence Interval	*p*-Value
Age			0.834			
Age < 65	Ref.					
65 ≤ Age ≤ 75	0.88	0.57–1.37				
Age > 75	0.95	0.63–1.44				
Previous TUR history	1.09	0.78–1.52	0.614			
Sex (Female)	0.94	0.60–1.48	0.802			
Previous BCG treatment history	1.22	0.76–1.94	0.414			
Upper Tract Urothelial Carcinoma history	1.36	0.92–2.04	0.143			
BCG dose reduction (half)	1.21	0.57–2.58	0.624			
Pathologic T stage (T1)	1.18	0.86–1.60	0.302			
Pyuria (more than W5)	1.47	1.08–2.00	0.013	1.31	0.95–1.80	0.095
Large tumour	1.51	1.11–2.06	0.009	1.36	0.98–1.87	0.063
Multiple tumour	1.59	1.13–2.24	0.008	1.51	1.07–2.15	0.019
Concomitant CIS	0.97	0.68–1.41	0.902			

**Table 4:** Univariate and multivariable cox proportional hazards regression for progression-free survival.

	Univariate			Multivariable		
Variables	Hazard Ratio	Confidence Interval	*p*-Value	Hazard Ratio	Confidence Interval	*p*-Value
Age			0.866			
Age < 65	Ref.					
65 ≤ Age ≤ 75	0.66	0.15–2.97				
Age > 75	0.81	0.20–3.25				
Previous TUR history	0.64	0.21–1.97	0.435			
Sex (Female)	0.49	0.06–3.80	0.498			
Previous BCG treatment history	1.62	0.36–7.33	0.529			
Upper Tract Urothelial Carcinoma history	0.56	0.07–4.31	0.578			
BCG dose reduction (half)	2.40	0.31–18.59	0.402			
Pathologic T stage (T1)	3.33	1.02–10.89	0.046			0.157
Pyuria (more than W5)	3.52	1.08–11.44	0.036	4.51	1.22–16.66	0.024
Large tumour	2.34	0.74–7.38	0.147			
Multiple tumour	6.32	0.82–48.93	0.078	5.97	0.77–46.28	0.087
Concomitant CIS	2.20	0.72–6.72	0.167			

## Data Availability

The data presented in this study are available on request from the corresponding author. The data are not publicly available due to patient privacy.
